# The Codon Usage Bias Analysis of Free-Living Ciliates’ Macronuclear Genomes and Clustered Regularly Interspaced Short Palindromic Repeats/Cas9 Vector Construction of *Stylonychia lemnae*

**DOI:** 10.3389/fmicb.2022.785889

**Published:** 2022-03-03

**Authors:** Ying Wang, Lin Yao, Jinfeng Fan, Xue Zhao, Qing Zhang, Ying Chen, Changhong Guo

**Affiliations:** ^1^Key Laboratory of Biodiversity of Aquatic Organisms, Harbin Normal University, Harbin, China; ^2^Key Laboratory of Molecular Cytogenetics and Genetic Breeding of Heilongjiang Province, Harbin, China; ^3^School of Civil and Environmental Engineering, Harbin Institute of Technology (Shenzhen), Shenzhen, China

**Keywords:** ciliates, macronuclear genome, codon usage bias, CRISPR/Cas9, optimizing vector

## Abstract

Ciliates represent higher unicellular animals, and several species are also important model organisms for molecular biology research. Analyses of codon usage bias (CUB) of the macronuclear (MAC) genome in ciliates can not only promote a better understanding of the genetic mode and evolution history of these organisms but also help optimize codons to improve the gene editing efficiency of model ciliates. In this study, macronuclear genome sequences of nine free-living ciliates were analyzed with CodonW software to calculate the following indices: the guanine-cytosine content (GC); the frequency of the nucleotides U, C, A, and G at the third position of codons (U3s, C3s, A3s, G3s); the effective number of codons (ENC); the correlation between GC at the first and second positions (GC12); the frequency of the nucleotides G + C at the third position of synonymous codons (GC3s); the relative synonymous codon usage (RSCU). Parity rule 2 plot analysis, neutrality plot analysis, and correlation analysis were performed to explore the factors that influence codon preference. The results showed that the GC contents in nine ciliates’ MAC genomes were lower than 50% and appeared AT-rich. The base compositions of GC12 and GC3s are markedly distinct and the codon usage pattern and evolution of ciliates are affected by genetic mutation and natural selection. According to the synonymous codon analysis, the codons of most ciliates ended with A or U and eight codons were the general optimal codons of nine ciliates. A clustered regularly interspaced short palindromic repeats/Cas9 (CRISPR/Cas9) expression vector of *Stylonychia lemnae* was constructed by optimizing the macronuclear genome codon and was successfully used to knock out the *Adss* gene. This is the first such extensive investigation of the MAC genome CUB of ciliates and the initial successful application of the CRISPR/Cas9 technique in free-living ciliates.

## Introduction

The genetic code is a set of rules for encoding information in DNA or mRNA sequences (Giulio, 1992). Codon usage bias (CUB) is widespread in genomes and has a profound impact on eukaryote genome evolution from yeast to *Caenorhabditis* and *Drosophila*, and eventually to humans (Sharp et al., 1995). Different species have diverse codons, and one type of codon has various frequencies in different species (Qin et al., 2005; Belalov and Lukashev, 2013; Yang et al., 2021). Investigations of CUB could provide a better understanding to the mechanism of underlying gene expression, molecular evolution, and host–pathogen coadaptation, and help predict the optimal codon among the highly expressed genes of a species (Henry and Sharp, 2007; Moura et al., 2011; Lal et al., 2016; Jeacock et al., 2018). Screening the optimal codon is helpful for constructing expression vectors and improving gene expression efficiency (Gurkan and Ellar, 2011; Konczal et al., 2019). The more widely used indicators are guanine-cytosine (GC) content, the effective number of codons (ENC), the correlation between GC at the first and second positions (GC12), the frequency of the nucleotides G + C at the third position of synonymous codons (GC3s), the relative synonymous codon usage (RSCU), and the frequency of the nucleotides U, C, A, and G at the third position of codons (U3s, C3s, A3s, and G3s) (Wang et al., 2016). Different methods have been chosen to measure the CUB in different organisms and discuss the effect of mutation and natural selection on shaping codon usage patterns.

Single-cell protozoa represent an early stage of biological phylogeny, and they have drastically changed our understanding of molecular evolution and become a research model organism of evolution and cellular mechanism (Pawlowski, 2014). Free-living ciliates are a group of higher protozoa. They have the unique feature of possessing two morphologically and functionally distinct nuclei: macronuclear (MAC) and micronuclear (MIC) (Herrick, 1994). The MAC has transcriptional activity and functions in all cellular events, while the MIC mainly acts during sexual reproduction (Orias, 1991). One of the key problems in gene engineering of ciliates is the regulation method of MAC gene expression, and the analysis of codon usage is an important aspect of MAC genome expression research.

In recent years, more and more research have focused on the MAC genome of ciliates (Bellec et al., 2014; Kumar and Kumari, 2015; Popenko et al., 2015; Iwamoto et al., 2018). The sequences of MAC genomes of nine type ciliate species have been published, thus providing a much stronger basis for the analysis of codon preference than previous (Doak et al., 2003; Aury et al., 2006; Eisen et al., 2006; Aeschlimann et al., 2014; McGrath et al., 2014; Xiong et al., 2015; Yi et al., 2016; Slabodnick et al., 2017; Zheng et al., 2018). The synonymous CUB of the MAC genomes in *Tetrahymena thermophila* and *Paramecium tetraurelia* has been reported and differences in the efficiency of translation of the reassigned stop codons and the possibility of translational efficiency between the two species were found (Salim et al., 2008). However, the general codon usage pattern of MAC genomes and the genetic mechanism in other ciliates have not been fully investigated.

In this study, we collected the MAC genome information of nine free-living ciliates from multiple databases, and a comprehensive analysis on the CUB of MAC genomes was performed to uncover codon usage pattern in ciliate MAC and identify the optimal codon. In addition, *Stylonychia lemnae*, a representative species of hypotrichous ciliates and an important model organism in pattern formation research, was selected to construct a clustered regularly interspaced short palindromic repeats/Cas9 (CRISPR/Cas9) expression vector based on the optimal codon to perform adenylosuccinate synthase (*Adss*) gene knockout.

## Materials and Methods

### Coding Sequence Data and Nucleotide Composition Analysis

The complete coding sequences (CDSs) of nine ciliates’ MAC genomes were retrieved from the National Center for Biotechnology Information^1^ and other ciliate databases (Table 1). Perl scripts were developed by our team to ensure that the CDSs had maximum applicability (Supplementary Material 1). Each CDS sequence should be more than 300 bp after deleting the Met (methionine), Trp (tryptophan), repeated sequences, and reverse complementary sequences. A stop codon in the middle of the sequence was deleted, and the start codon ATG and the end stop codons TAA, TAG, and TGA are retained. Only perfect CDSs with an exact multiple of three bases and correct start and stop codons were analyzed (Wright, 1990).

**TABLE 1:** General information of nine ciliates’ MAC genomes.

Number	Class	Species	Database information
1	Oligohymenophorea	*Tetrahymena thermophila*	http://ciliate.org/index.php/home/welcome
2	Oligohymenophorea	*Paramecium caudatum*	https://paramecium.i2bc.paris-saclay.fr/
3	Oligohymenophorea	*Paramecium tetraurelia*	https://paramecium.i2bc.paris-saclay.fr/
4	Oligohymenophorea	*Pseudocohnilembus persalinus*	NCBI Datasets–Genomes
5	Heterotrichea	*Stentor coeruleus*	http://stentor.ciliate.org/index.php/home/welcome
6	Spirotrichea	*Oxytricha trifallax*	http://oxy.ciliate.org/index.php/home/welcome#
7	Spirotrichea	*Stylonychia lemnae*	http://stylo.ciliate.org/index.php/home/welcome
8	Spirotrichea	*Uroleptopsis citrina*	NCBI Datasets–Genomes
9	Spirotrichea	*Euplotes octocarinatus*	http://evan.ciliate.org

The nucleotide contents of nine ciliates’ MAC genomes were calculated by CodonW software, including the overall nucleotide compositions (A, C, U, and G%), GC content, the nucleotide compositions at the third position (A3s, U3s, C3s, and G3s), G + C% at the first (GC1), the second base of codon (GC2), the third base of codon (GC3), and mean nucleotides G + C% at the first and second positions (GC12).

### Effective Number of Codons

The ENC was used to quantify the CUB in one specific gene (Wright, 1990). ENC-GC_3_ was used to analyze the influence of the GC_3_ content on codon usage, the expected ENC value for each GC_3_ was calculated using the following formula (Booth et al., 2015), and the expected fitting curve of ENC values was drawn with GraphPad Prism 8.0.^2^


$$E\,N\,C_{e\,x\,p\,e\,c\,t\,e\,d}=2+G\,C_{3}+29/\left[G\,C_{3}^{2}+{\left(1-G\,C_{3}\right)}^{2}\right]$$

### Parity Rule 2 Plot Analysis

Parity rule 2 (PR2) was used to analyze the impact of mutational pressure and selective constraints on the CUB of a gene. The PR2 plots were generated by GraphPad Prism 8.0. The ordinate of the plots was AT bias at the third base [A3/(A3 + T3)], and the abscissa was GC bias at the third base [G3/(G3 + C3)]. The center was 0.5, with A = T and G = C, which means that no bias occurred between the two strands of DNA for mutation and selection rates (Moradian et al., 2007).

### Neutrality Plot Analysis

The neutrality plot was used to explore the effect of mutation and selection on CUB by regression of GC12 on GC3s. Neutrality plots were drawn by GraphPad Prism 8.0, GC3s represented the abscissa, and GC12 represented the ordinate. When the slope of the regression line is close to 1, mutation pressure might be the main force for CUB, whereas when the slope is 0, natural selection is the dominant force. A slope of ± 0.5 indicates no or weak external selection pressure (Sueoka, 1993).

### Relative Synonymous Codon Usage

Relative synonymous codon usage is the ratio of the observed frequency of a specific codon to the expected value. It was calculated using CodonW software. If all synonymous codons are used equally, then the RSCU values are close to 1.0 (Gupta et al., 2004).

### Correspondence Analysis

Multivariate statistical analysis was conducted on the codons (A3s, C3s, U3s, G3s, ENC, etc.) to examine the variations in CUB in nine ciliates. The average values were calculated using IBM SPSS Statistics 20 to test the correlation between codon usage variation and base composition.

### Clone the *Adss* Gene of *Stylonychia lemnae*

Primers were designed according to the sequence of the adenylosuccinate synthase (*Adss*) gene (Contig2018.g2178) in the *S. lemnae* genome database.^3^

Total RNA of *S. lemnae* was extracted using an RNA kit (Tiangen, Beijing, China) in accordance with the manufacturer’s method. The quality and purity of total RNA were further assessed with 1% agarose gel electrophoresis. Approximately 1,000 ng of total RNA was reverse transcribed into cDNA using the Prime Script RT Reagent Kit (TransGene, Beijing, China). The amplification procedure was conducted as follows: 95°C for 5 min predenaturation, followed by 40 cycles at 95°C for 30 s for denaturation, 52°C for 30 s, 72°C for 30 s for annealing and extension (30 cycles), and 72°C for 10 min. The RT-PCR products were separated with 1.0% agarose gel electrophoresis.

### *Stylonychia lemnae* Clustered Regularly Interspaced Short Palindromic Repeats/Cas9 Expression Vector Construction

All-in-one-CRISPR/Cas9-LacZ, MLM3636, and PBR322 vectors were purchased from Addgene^4^ and Solarbio.^5^ These vectors were constructed as follows:

#### Target Selection for Single Guide RNA

The specificity of the Cas9 nuclease was determined by the 20-nt guide sequence within single guide RNA (sgRNA). sgRNA was designed through an online website (Zhang Feng Lab^6^) according to the characteristics of the *Adss* gene sequence and the design principles, and it was amplified by PCR with sgRNA-F/sgRNA-R primers (Supplementary Table 1); the sgRNA was recombined into the target vector MLM3636 by the recombination method. The connection reaction included 5 μl PCR-amplified fragments, 1 μl vector after digestion, 10 μl recombinase, and 4 μl nuclease-free water in a 20-μl total volume.

#### All-in-One-Clustered Regularly Interspaced Short Palindromic Repeats/Cas9-LacZ Vector Optimization

The mutation PCR conditions were as follows: 1 μl all-in-one-CRISPR/Cas9-LacZ vector, 1 μl Mutation-S, 1 μl Mutation-A, 25 μl 2× TransStarl FastPfu PCR SuperMix, and 22 μl nuclease-free water in a 50-μl total volume. The PCR conditions were as follows: denaturation at 95°C/3 min, annealing at 60°C/20 s, and 35 cycles of 72°C/5 min. Ten microliters of PCR product was used for agarose gel electrophoresis detection, and the remaining 40 μl of PCR product was digested with 1 μl DMT enzyme for 1 h (Supplementary Table 1).

#### The Expressing Vector Construction

The successfully mutated mAll-in-one-CRISPR/Cas9-LacZ (or All-in-one-CRISPR/Cas9-LacZ) and PBR322 vectors were simultaneously digested with *Sal*I and *Nru*I, ligated with the T_4_ DNA enzyme (TransGen Biotech, Beijing, China), then transformed and verified. Finally, mPBR322-All-in-one-CRISPR/Cas9-LacZ and PBR322-All-in-one-CRISPR/Cas9-LacZ vectors were obtained. The enzyme cut system was as follows: 1 μl mAll-in-one-CRISPR/Cas9-LacZ (or All-in-one-CRISPR/Cas9-LacZ/PBR322 vector)/PBR322 vector, 5 μl 10× Cutsmart buffer, 1 μl *Sal*1-HF, 1 μl *Nru*1-HF, and 12 μl nuclease-free water in a 20-μl total volume. Incubate at 37°C for 15 min and then gel to recover the target fragments. The connection reaction conditions were as follows: 2 μl 5× T_4_ DNA buffer, 0.5 μl T_4_ DNA ligase, 3 μl enzyme digestion target fragment, 0.5 μl restriction digestion vector fragment, and 13 μl nuclease-free water in a 20-μl total volume. The recombination vectors were incubated for 2 h at 25°C for transformation into competent cells. Positive cells were picked, vectors were extracted for electrophoresis detection, and the Harbin Boss Biological Company was used for sequencing. Then, the original vector sequence results were compared with the mutated vector sequence results. The ligation results of the all-in-one-CRISPR/Cas9-LacZ and PBR322 vectors showed that the length of target band was greater than 10,000 bp; thus, the correct recombinant vector was obtained (Supplementary Figure 1).

### Detection and Quantitative Real-Time PCR Testing

The co-transformation of mPBR322-All-in-one-CRISPR/Cas9-LacZs (or PBR322-All-in-one-CRISPR/Cas9-LacZs) and MLM3636-sgRNA vectors was conducted in *S. lemnae* by feeding method, and the green fluorescent protein was a marker to be observed by fluorescence microscopy (Axio Imager A2). The positive cells with changes were selected for quantitative real-time PCR (qRT-PCR), and 18s rRNA was used as the endogenous reference gene for normalization (based on the manufacturer’s instructions for the single cell sequence specific amplification kit and ChamQTM Universal SYBR Steps for qRT-PCR Master Mix kit).

## Results

### Codon Base Composition

CodonW software was used to analyze the base composition of the eligible coding DNA sequence in the MAC genomes of the nine ciliates (Table 2). The GC contents of the MAC genomes ranged from 18.19 to 40.19%. All values were less than 50%, which indicated that these MAC genomes were all AT-rich. Diverse GC contents were observed at different positions of the codons. The GC12 content ranged from 11.30 to 65.90%, with an average value of 18.74∼40.80%. The GC3s content ranged from 4.30 to 81.50%, with an average value of 17.63∼39.58%. These results showed that the GC contents at the third position of the codon varied more than those of the first and second positions. The GC3s content of the four species in Spirotrichea ranged from 23.26 to 39.58%, that of *Stentor coeruleus* was 28.31, and that of the four species in Oligohymenophorea ranged from 17.63 to 26.50%. The average contents of GC12 and GC3s in *P. tetraurelia* and *Paramecium caudatum* were closer than that of the other species. Thus, the closer the genetic relationship, the more similar the GC content.

**TABLE 2:** GC content of nine ciliates’ codons.

Species	GC content (%) in MAC genomes				
	GC12 content range	GC12 average content	GC3s content range	GC3s average content	GC average content
*S. lemnae*	20.25∼62.20	34.39	14.60∼78.40	29.58	21.33
*T. thermophila*	11.30∼59.00	28.89	6.40∼71.20	21.67	25.28
*O. trifallax*	21.90∼53.90	34.14	14.60∼73.20	31.11	32.63
*P. tetraurelia*	12.40∼48.20	27.96	4.50∼51.50	26.50	27.23
*P. caudatum*	12.90∼47.40	27.39	4.30∼49.80	25.50	26.45
*E. octocarinatus*	31.30∼44.90	37.63	25.70∼46.20	23.26	30.45
*S. coeruleus*	19.70∼65.90	29.50	14.80∼77.10	28.31	28.91
*U. citrina*	23.70∼60.70	40.80	9.10∼81.50	39.58	40.19
*P. persalinus*	13.60∼25.10	18.74	11.30∼34.90	17.63	18.19

### Synonymous Codon Usage

**FIGURE 1:**
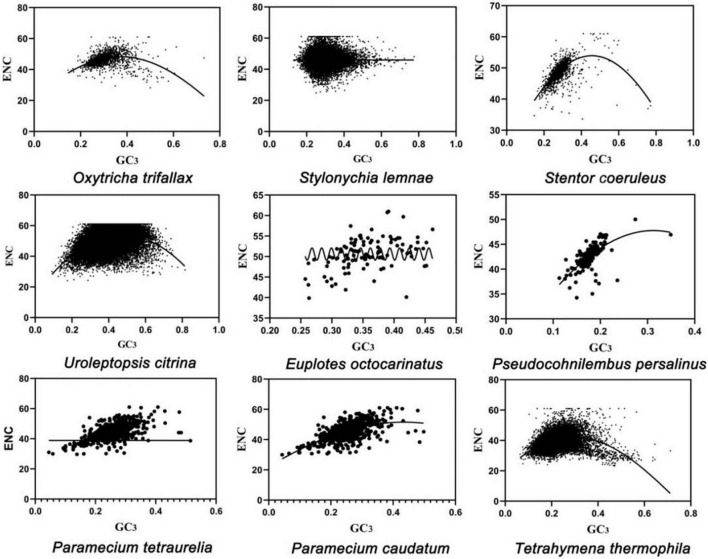
Effective number of codons (ENC) plot analysis of nine ciliates. Abscissa: GC_3_, GC content of the third base. Ordinate: ENC, number of effective codons.

An ENC-GC_3_ association analysis was performed to explore the relationship between codon preference and base composition (Figure 1). The distribution of the ENC ratio frequency of the nine ciliates (Supplementary Table 2) and ENC values (Supplementary Table 3) showed that the actual ENC ratios of the nine ciliates were in the expected ratio range and greater than 0.5. Therefore, most genes were closer to the standard curve and suggested that gene mutation was the main factor in the formation of codon preference of the ciliates’ MAC genomes. However, most of ENC values were between 45 and 61 in seven species and distributed on both sides of the standard curve. This result indicated that the codon preference of the ciliates’ MAC genomes was low. Only ENC values of *T. thermophila* and *Pseudocohnilembus persalinus* in Oligohymenophorea were between 35 and 45, which showed a relatively high codon preference of the two species.

**FIGURE 2:**
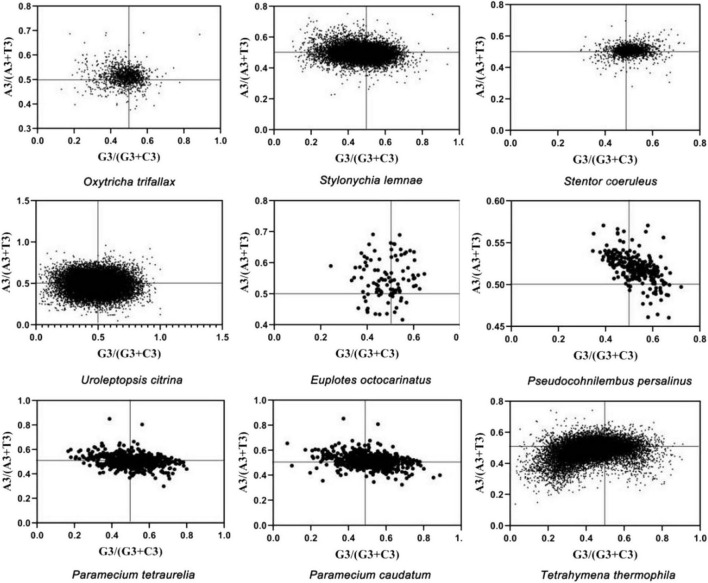
Parity rule 2 (PR2)-bias plot analysis of nine ciliates. Abscissa: [G3/(G3 + C3)], GC bias in the third codon position. Ordinate: [A3/(A3 + T3)], AT bias in the third codon position.

For the bias analysis, the PR2 bias plot was drawn with A3/(A3 + T3) as the ordinate and G3/(G3 + C3) as the abscissa (Figure 2), and the average distribution of genes was calculated to evaluate the relationship between purine and pyrimidine in each gene codon. Results revealed that the third base of the codons in ciliates’ MAC genomes had different usage bias. *P. tetraurelia* and *P. caudatum* tended to use T and G, and *S. coeruleus* showed a significant tendency to use T and G. The patterns of the codon usage of *Euplotes octocarinatus* and *P. persalinus* were similarly biased to use A and G. *T. thermophila*, *S. lemnae*, and *Uroleptopsis citrina* preferred to use T and C in the third position of the codon. *Oxytricha trifallax* preferred to end with A and C. These results suggested that the codon preferences of the ciliates’ MAC genomes were affected by both selection pressure and multiple factors.

**FIGURE 3:**
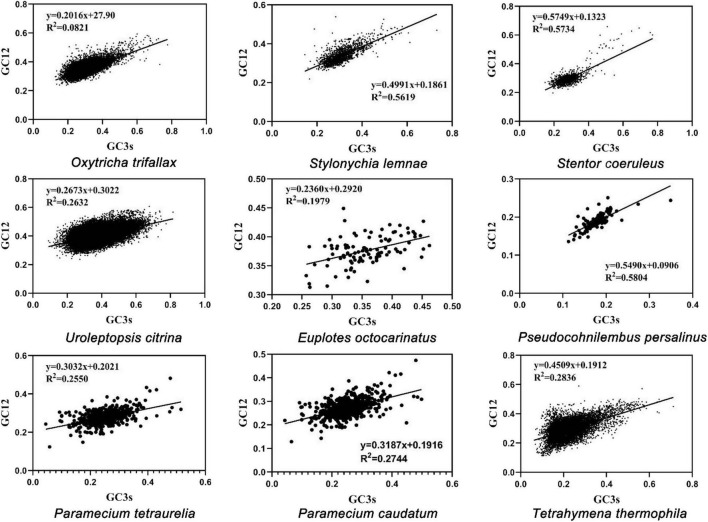
Neutrality plot analysis of nine ciliates. Abscissa: GC3s, G + C content at the third position of synonymous codons. Ordinate: GC12, GC content in the first and second positions of the codons (GC1 and GC2).

The neutrality plot was analyzed using the Pearson correlation coefficient of GC12 and GC3s (Figure 3). The correlation coefficients of GC12 and GC3s in MAC genomes of nine ciliates ranged from 0.445 to 0.781, showing extremely significant positive correlation. The regression coefficient ranged from 0.2016 to 0.5749. These results showed that the codon preferences of the nine ciliates’ MAC genomes were mainly determined by gene mutations. According to the *K*-value of the regression curve, *O. trifallax* was least affected by genetic mutations and *P. persalinus* was most affected. The degree of genetic mutation had a similar influence on *P. tetraurelia* and *P. caudatum*.

### Determination of Optimal Codons by Relative Synonymous Codon Usage

**FIGURE 4:**
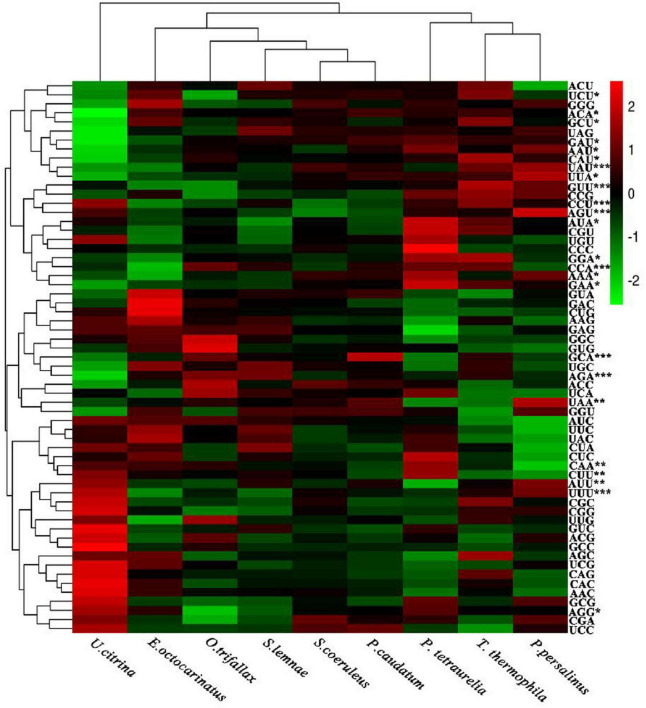
Heatmap of relative synonymous codon usage (RSCU) values of nine ciliates. *** represents the codon commonly preferred by nine ciliates; ** represents the codon commonly preferred by the four ciliates of Spirotrichea; * represents the codon commonly preferred by the four ciliates of Oligohymenophorea.

The numerical value of RSCU was used as the standard to select the most preferred codon. The 10% of genes at each end were chosen to build two databases of the high and low biases. Codons with ΔRSCU > 0.08 in the two databases were considered (Figure 4 and Supplementary Table 4). ΔRSCU values of eight codons UUU, UAU, CCU, CCA, GUU, GCA, AGA, and AGU in the nine ciliates’ MAC genomes were greater than 0.08, indicating that these codons were the optimal codons and commonly preferred in the nine ciliates. Moreover, these codons encoded seven amino acids Phe, Tyr, Pro, Val, Ala, Arg, and Ser. Pro had two optimal codons CCU and CCA. The commonly preferred codons of the four species in Spirotrichea were AUU, UAA, CAA, and CUU, which encoded Ile, Gln, and Leu. Gln had two optimal codons UAA and CAA. The commonly preferred codons of the four species in Oligohymenophorea were AAA, GAU, CAU, AAU, GAA, AUA, GGA, ACA, GCU, AGG, UUA, and UCU, which encoded the 12 amino acids Lys, Asp, His, Asn, Glu, Ile, Gly, Thr, Ala, Arg, Leu, and Ser, respectively. Unlike Oligohymenophorea and Spirotrichea, the preferred codons of Heterotrichea were UGU, GGU, and GUA, which encoded Cys, Gly, and Val, respectively.

### Correlation Analysis

To further determine the preferred codon of the nine ciliates, a correlation analysis was performed among the third bases of the synonymous codons of the ciliates (Supplementary Table 5). The results showed that the GC3s, T3s, C3s, A3s, and G3s of *O. trifallax*, *U. citrina*, *E. octocarinatus*, *P. persalinus*, *P. tetraurelia*, *P. caudatum*, and *T. thermophila* were extremely and significantly correlated. The GC3s of *S. lemnae* was very significantly correlated with C3s and A3s. The GC3s of *S. coeruleus* was very significantly correlated with C3s, A3s, and G3s. Except for *S. lemnae*, the ENC values of the remaining eight ciliates were extremely significantly correlated with the total GC content at the three positions of the codon. The correlations between the ENC and T3s, C3s, A3s, and G3s of *E. octocarinatus*, *S. coeruleus*, and *U. citrina* reached highly significant levels.

### *Adss* Gene of *Stylonychia lemnae*

*Adss* is a purine ribonucleoside monophosphate that exists widely in human and animal cells and plays an important role in the metabolism of nucleotide cycles. The *S. lemnae Adss* gene was significantly upregulated by hEGF induction and associated with several cell division genes (Mu et al., 2016). According to the transcriptome data of *S. lemnae*, the estimated length of the *Adss* gene was approximately 1,000 bp. This length was suitable for the CRISPR/Cas9 experiment.

To determine the gene sequence, *S. lemnae* total RNA was reverse transcribed into cDNA and then PCR amplification was performed using cDNA as a template to obtain the *Adss* gene. Electrophoresis results showed that the size of the amplified product was approximately 1,300 bp, which was consistent with the size of the target gene (Supplementary Figure 2A).

### Vector Mutation and Construction

The upstream and downstream sequences were identified through high specificity in the exons following the first start codon ATG. The target sequence of the *S. lemnae Adss* gene has fifteen sites that meet the requirements of a 5′-NGG PAM (Porto-spacer motif) and contains three GGG codons, three TGG codons, and nine AGG codons. According to the design principles of the sgRNA sequence, we chose 5′-CTACATCAAGTTCATCGAAAAGG-3′, which preceded a 5′-AGG PAM, and approximately 20-nt guide sequence base pairs with the opposite strand to mediate Cas9 (Supplementary Figure 2B).

Furthermore, we optimized the gene knockout vector of Cas9 by gene mutation to reduce the probability of off-targeting. With the mutation primers, we tried to optimize the triplet codons of *S. lemnae*, and the GGU at position 34 in the All-in-one-CRISPR/Cas9-LacZ vector was successfully mutated to GGA. The results were verified by MEGA and Next-Generation Sequencing (Supplementary Figure 3).

Double enzyme digestion was performed to test whether the vector had been connected in a directional manner. The restriction enzyme fragments of the PBR322-All-in-one-CRISPR/Cas9-LacZ and mPBR322-All-in-one-CRISPR/Cas9-LacZ vector were both 7,681 and 3,013 bp (Supplementary Figure 4A), while the non-enzymatic fragment of the All-in-one-CRISPR/Cas9-LacZ and PBR322 vectors was approximately 10,000 bp, which was almost the sum of them (Supplementary Figure 4B).

### Validity of Gene Knockout

The mPBR322-All-in-one-CRISPR/Cas9-LacZ (or PBR322-All-in-one-CRISPR/Cas9-LacZ) and MLM3636-sgRNA vectors were transfected into *S. lemnae*, which was targeted to the specific sequence of the *Adss* target gene to knock it out. After 24 h, the status of *S. lemnae* was observed by fluorescence microscopy (Table 3). Group 1 and Group 2 showed that the mutated vector had a more significant effect than the unmutated vector on the movement of *S. lemnae*. Furthermore, the effects of the two groups both developed after 48 h (Group 3 and Group 4). This proved that the optimized vectors according to the best codon of *S. lemnae* are efficient.

**TABLE 3:** Effects of transfection with different vectors on *Stylonychia lemnae*.

Vectors	Treating time (h)	Effects
Group 1: mutated Cas9 expression vector + sgRNA expression vector	24	Total loss of movement
Group 2: unmutated Cas9 expression vector + sgRNA expression vector	24	Slow motion
Group 3: mutated Cas9 expression vector + sgRNA expression vector	48	Death
Group 4: unmutated Cas9 expression vector + sgRNA expression vector	48	Slow motion

**FIGURE 5:**
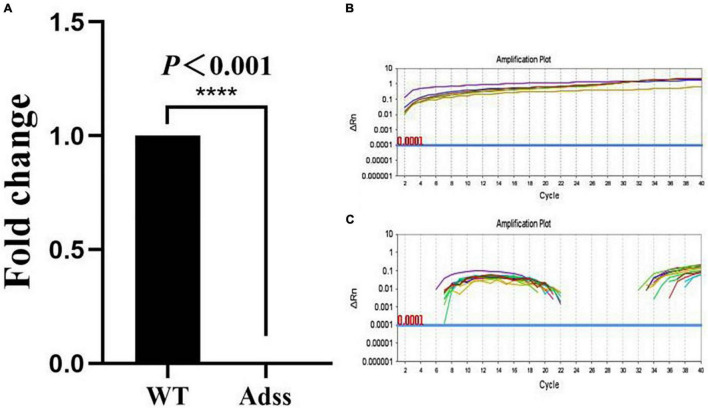
Quantitative analysis of expression of *Adss* gene expression in the wild-type cells and gene-knockout cells of *Stylonychia lemnae*. **(A)** Expression of the *Adss* genes in wild-type *S. lemnae* and gene-knockout *S. lemnae*. **(B)** Expression curve of wild-type cells. **(C)** Expression curve of *Adss* gene-knockout cells. WT, wild-type; Adss, adenylosuccinate synthase. ****P < 0.001, the difference is highly significant.

The expression level of the *Adss* gene was detected by qRT-PCR in both the wild-type individuals and knockout individuals of *S. lemnae* (Figure 5). The results showed that the *Adss* gene was expressed normally in wild-type *S. lemnae* (Figures 5A,B), although most fragments of the *Adss* gene were missed in knockout cells (Figures 5A,C). The aforementioned results showed that the *Adss* gene can be knocked out by the optimized knockout vector.

## Discussion

### Patterns of Macronuclear Genome Codon Usage Bias in Free-Living Ciliates

The GC contents of the whole MAC genomes in nine free-living ciliates ranged from 18.19 to 40.19%, indicating that these MAC genomes were all AT-rich. At the same time, the GC12 analysis showed that the GC contents at the first and second codon positions were higher than that of GC3s in nine ciliates’ MAC genomes (Table 2). A subset of highly expressed genes to analyze CUB in *Tetrahymena* found a strong bias toward codons with low GC, and termination codons were always AT-rich, which was consistent with the results in our study (Salim et al., 2008). The RSCU results showed that the nine MAC genomes preferred codons ending in U or A to those endings in C or G (Figure 4). A correlation analysis between the ENC and T3s, C3s, A3s, G3s, GC3s, and GC12 indicated that the base content at the first, second, and third positions of the synonymous codons directly affect the level of CUB (Supplementary Table 5). The RSCU analysis revealed eight common codon endings with U and/or A, and eight codons were observed more frequently than expected (Figure 4). This finding supports a high degree of uniformity between the closely related species.

To investigate the influence factors of CUB, the ENC values of each protein-coding gene expressed in nine ciliates’ MAC were plotted. The standard curve showed that CUB was mainly due to mutation in GC_3_ (Figure 1). PR2-bias plots suggested that mutation was not the only affecting factor on CUB because the centers of the data distribution were not evenly distributed in the plot (Figure 2). The neutral correlation analysis established the relationship between GC12 and GC3s to show the role of natural selection or mutation in shaping the CUB based on the *K*-value among the nine ciliate species (Figure 3). All of the aforementioned results suggested that the selection–mutation–drift theory could be used to explain most of the CUB in the nine ciliates’ MAC genomes and mutation was dominant in specific species, while natural selection appeared to have a greater effect at the class level. In previous research, selection–mutation–drift theory has been used to explain CUB in all kinds of organisms, and the prevailing view was that the direction of genetic mutations was affected by natural selection (Bulmer, 1991; Akashi, 1995; Akashi and Schaeffer, 1997; Akashi and Eyre-Walker, 1998; Shah and Gilchrist, 2011). This hypothesis indicates that natural selection primarily operates to improve replication fidelity, although with ultimate limits regarding what can be achieved based on the power of random genetic drift. Moreover, species-specific variation in the production of mutator and/or antimutator alleles may be an additional contributor (Lynch et al., 2016).

Previous studies on codons in ciliates were mainly based on the mitochondrial genome, partial genomic data, deviations in the nuclear genome, specificity of stop codon usage, and environmental adaptation (Kim et al., 2005; Swart et al., 2012; Sheng et al., 2020; Arella et al., 2021). This paper is the first analysis of MAC genome CUB in so many species of ciliates and demonstrated that species surviving in more similar environments show a greater likelihood of presenting similar codon usage patterns. However, the mechanisms responsible for the discontinuity in scaling the mutation rate with genome size remain unclear. To enhance the understanding of the codon bias within and between genomes, it should be conducted in entire genomics system rather than in individual genes or collections of genes (Plotkin and Kudla, 2011).

### Phylogenetic Analysis Based on Relative Synonymous Codon Usage

For ciliates, previous research on phylogeny of CUB were based on different gene sequences and the conclusions were hard to be unified (Tourancheau et al., 1995; Lozupone et al., 2001; Barth and Berendonk, 2011; Heaphy et al., 2016). These nine free-living ciliates were mainly divided into three classes based on SSU rDNA sequences (Supplementary Figure 6 and Supplementary Table 6) according to traditional phylogenetic tree of ciliates (Gao et al., 2009, 2016; Li et al., 2010; Sun et al., 2010; Zhan et al., 2013; Huang et al., 2014; Lu et al., 2019). The phylogenetic tree based on RSCU of MAC genomes illustrated a completely different topology (Supplementary Figure 5). In particular, two species of *Paramecium* were separated far away, which is the same as the result in RSCU of mitochondrial genome (Barth and Berendonk, 2011). It is believed that there is an independent evolutionary mechanism for CUB, but the research on evolution mechanism of CUB in different organisms is far from enough.

### Clustered Regularly Interspaced Short Palindromic Repeats/Cas9 Vector Based on MAC Genomes’ Optimal Codon of Ciliates

CRISPR/Cas9 is an efficient and powerful tool for ciliate molecular biology research. However, the scope of the editing system is limited by the PAM site NGG, and there are still no successful cases in free-living ciliates.

In this study, based on the optimal codons of ciliates’ MAC genomes, we constructed a CRISPR/Cas9 gene knockout vector of *S. lemnae* by mutation of GGU encoding glycine to GGA to improve the expression efficiency of the vector encoding Cas9. This optimized CRISPR/Cas9 vector was used for *Adss* gene knockout and a significant effect on cells was observed at the cytology level. Results of qRT-PCR showed that the *Adss* gene has been knocked out successfully from the positive cells (Table 3 and Figure 5).

The optimal codon screening based on multiple related species is obviously better than single species to obtain the general optimal codon of ciliates. However, due to the limitation of available MAC genome data, the genetic relationships of nine species are distant, and eight general codons in this study can only reflect the main tendency of optimal codon usage of ciliates’ MAC. The general codes for more closely related species are more useful in gene editing. With increases of ciliates’ MAC genome information in the future, we can obtain more general codons to improve the efficiency of ciliate gene editing research.

## Conclusion

The present study investigated the CUB in the MAC genomes of nine free-living ciliates by comprehensive analyses, and then the CUB pattern of these ciliates was illustrated. These macronuclear genomes were all AT-rich, the average content of GC3_*S*_ varied more than GC12, and most of these codons ended with A or U. The ENC-GC_3_, PR2-bias plot, and neutrality plot analysis suggested that selection–mutation–drift theory can be used to explain the formation mechanism of ciliates’ CUB, and mutation appeared to have a greater impact on the species level than natural selection. The pattern of CUB was similar in closely related species. The MAC genomes of nine free-living ciliates shared eight general optimal codons, UUU, UAU, CCU, CCA, GUU, GCA, AGA, and AGU. Based on the principle of general optimal codons, we constructed an optimized CRISPR/Cas9 expression vector of *S. lemnae* and successfully knocked out the *Adss* gene. This is the first study to analyze codon usage patterns based on the MAC genomes in such extensive species of free-living ciliates, and the CRISPR/Cas9 technique was successfully applied in free-living ciliates initially. The aforementioned results should be valuable for understanding the genetics and evolutionary mode of ciliates and investigating the gene function of model ciliates.

## Data Availability Statement

The original contributions presented in the study are included in the article/Supplementary Material, further inquiries can be directed to the corresponding authors.

## Author Contributions

YW and LY: methodology, investigation, data analysis, and writing—original draft. JF: methodology, investigation, and data analysis. XZ and QZ: methodology and investigation. CG: methodology and revision. YC: conceptualization, methodology, investigation, formal analysis, and writing—original draft and revision. All authors have agreed to authorship and the submission of this article for peer review.

## Conflict of Interest

The authors declare that the research was conducted in the absence of any commercial or financial relationships that could be construed as a potential conflict of interest.

## Publisher’s Note

All claims expressed in this article are solely those of the authors and do not necessarily represent those of their affiliated organizations, or those of the publisher, the editors and the reviewers. Any product that may be evaluated in this article, or claim that may be made by its manufacturer, is not guaranteed or endorsed by the publisher.

## Footnotes

1
http://www.ncbi.nlm.nih.gov

2
https://www.graphpad-prism.cn

3
http://stylo.ciliate.org/index.php/home/welcome

4
http://www.addgene.org/

5
http://www.solarbio.com/

6
http://crispr.mit.edu/
