# Supplementary material for: The Codon Usage Bias Analysis of Free-Living Ciliates’ Macronuclear Genomes and Clustered Regularly Interspaced Short Palindromic Repeats/Cas9 Vector Construction of Stylonychia lemnae
Source: Front Microbiol. 2022 Mar 3;13:785889. doi: 10.3389/fmicb.2022.785889 (PMC8927777; doi:10.3389/fmicb.2022.785889)
Supplement: Supplementary file 1 [file Data_Sheet_1.DOCX]

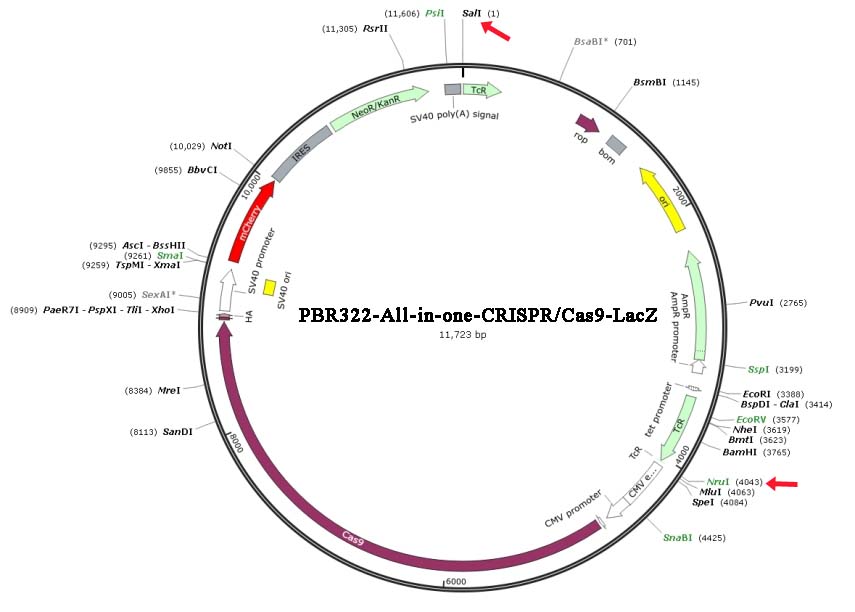


**FIGURE S1** PBR322-All-in-one-CRISPR/Cas9-LacZ vector map. Red arrows represent enzyme cut sites.


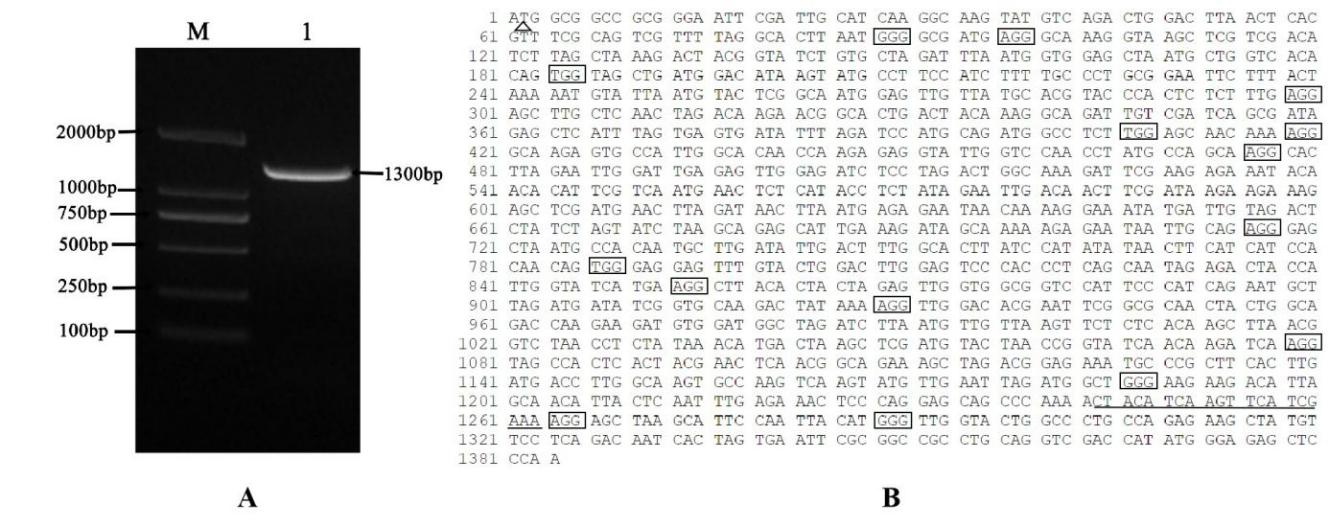


**FIGURE S2** Identification of *S. lemnae Adss* gene. **(A)** Electrophoresis of full-length target gene RT-PCR product. M, DL2000 DNA Marker; 1, *Adss* amplified by primers *Adss*-F/*Adss*-R. **(B)** *S. lemnae Adss* cDNA sequencing result. The horizontal line shows the sgRNA; the triangle shows the initiation codon ATG; the boxes show PAM sites.


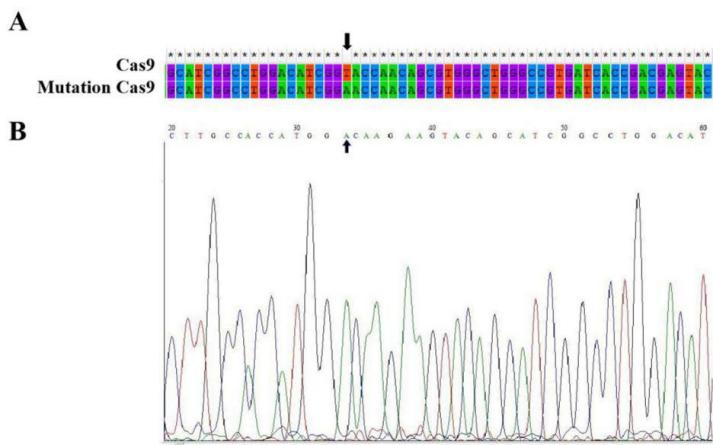


**FIGURE S3** The mutation result of all-in-one CRISPR/Cas9-LacZ vector. **(A)** MEGA sequence blast. Black arrows indicate the mutated base. **(B)** Next-Generation sequencing peaks figure of mutation vector sequence.


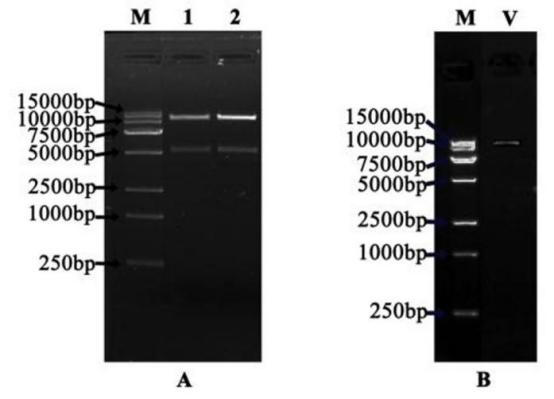


**FIGURE S4** Identification of restriction enzyme digestion of two vectors and the recombinant vector PCR product. **(A)** Two vectors digested by *Sal*I and *Nru*I. **(B)** Identification of the recombinant expression vector. M, DL15000 DNA Marker; 1, Original vector; 2, Optimized vector; V, Recombinant vector.


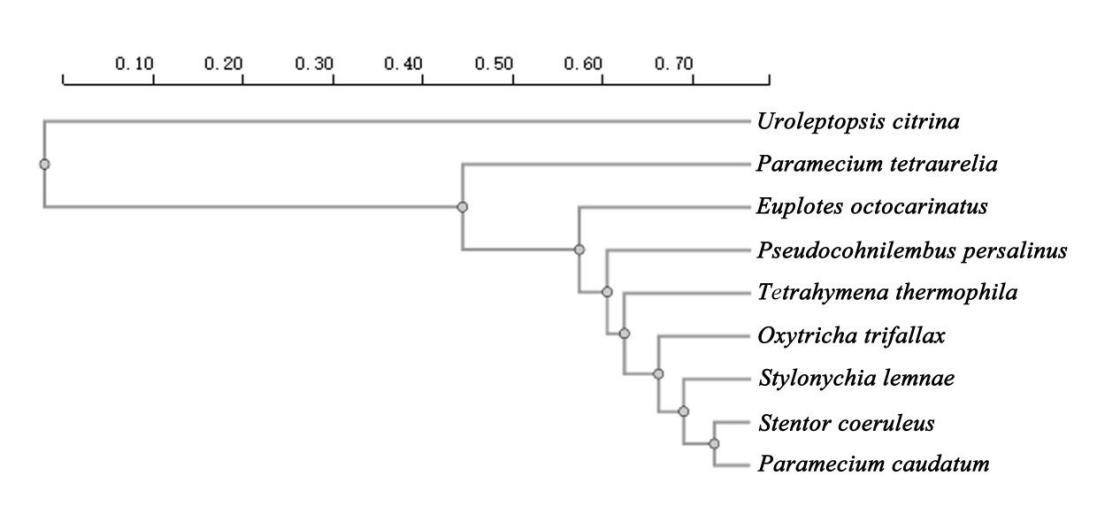


**FIGURE S5** Phylogenetic tree based on RSCU.

**
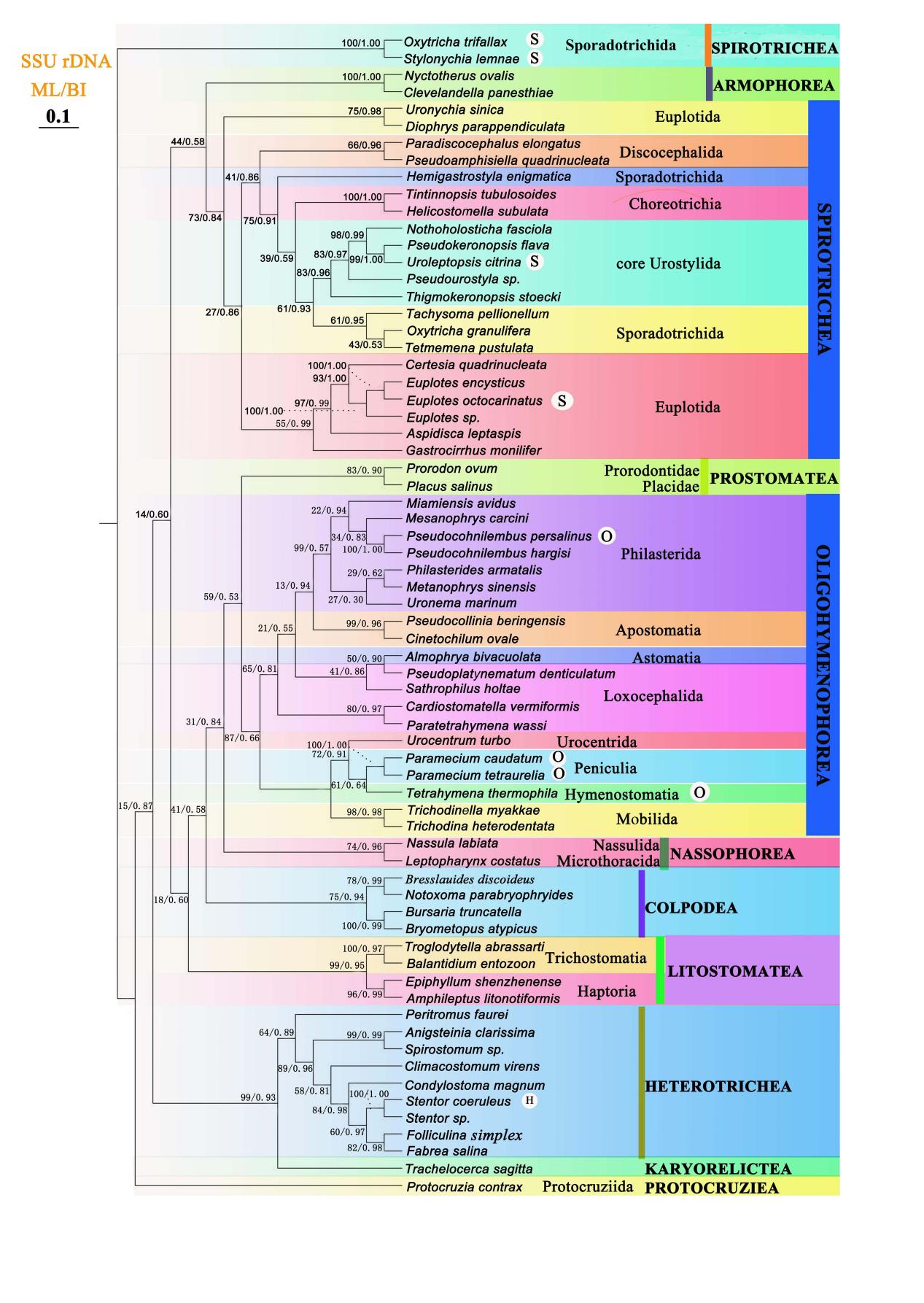
**

**FIGURE S6** Maximum likelihood (ML) phylogenetic tree of the phylum Ciliophora based on the SSU rDNA (68 taxa). Numbers at nodes represent the bootstrap values of maximum likelihood (ML) out of 1000 replicates and the posterior probability of Bayesian analysis (BI). The letters in the white circle represent different taxa:

H, Heterotrichea; O, Oligohymenophorea; R, Spirotrichea.

**TABLE S1 Primers used in this study.**

| **Primer name** | **Sequence information** |
| --- | --- |
| *Adss*-F(RT-PCR) | GTCTGAGGAACATAGCT |
| *Adss*-R(RT-PCR) | GCATCAAGGCAAGTA |
| 18s-F(qRT-PCR) | AAGAACGGCCATGCACCACC |
| 18s-R(qRT-PCR) | TTCAGCATCTTCCGAGAAATCAAAGT |
| *Adss*-F(qRT-PCR) | GGGAGCGAATGCTACAATGC |
| *Adss*-R(qRT-PCR) | TTCTGATGGGAATGGACCGC |
| Mutation-S | GACATCGGAACCAACAGCGTGGGCT |
| Mutation-A | GTTGGTTCCGATGTCCAGGCCGATG |
| sgRNA-F | GCAGGCTTTAAAGGAACCAATTCAGTCGACTGGATCCGGTA |
| sgRNA-R | GTAGAGAGGTACCTCGAGCGGCCCAAGCTTAAAAAAAGCACCGACTCGG |

**TABLE S2 ENC ratio of nine ciliates.**

| Species | Class boundary | Median | Amount | Frequency |
| --- | --- | --- | --- | --- |
| *S. lemnae* | -23.0～-5.0 | -14 | 1781 | 0.087 |
|  | -5.0～5.0 | 0 | 16605 | 0.813 |
|  | 5.0～17.0 | 11 | 2036 | 0.100 |
| *T. thermophila* | -17.0～-5.0 | -11 | 2105 | 0.082 |
|  | -5.0～5.0 | 0 | 21460 | 0.837 |
|  | 5.0～23.0 | 14 | 2081 | 0.081 |
| *O. trifallax* | -19.0～-5.0 | -12 | 130 | 0.088 |
|  | -5.0～5.0 | 0 | 1197 | 0.810 |
|  | 5.0～15.0 | 10 | 150 | 0.102 |
| *P. tetraurelia* | -17.0～-5.0 | -11 | 85 | 0.117 |
|  | -5.0～5.0 | 0 | 553 | 0.762 |
|  | 5.0～17.0 | 11 | 88 | 0.121 |
| 1. *caudatum* | -17.0~-5.0 | -11 | 107 | 0.138 |
|  | -5.0～5.0 | 0 | 571 | 0.736 |
|  | 5.0～17.0 | 11 | 98 | 0.126 |
| *E. octocarinatus* | -11.0～-5.0 | -8 | 11 | 0.108 |
|  | -5.0～5.0 | 0 | 85 | 0.833 |
|  | 5.0～11.0 | 8 | 6 | 0.059 |
| *S. coeruleus* | -17.0～-5.0 | -11 | 107 | 0.041 |
|  | -5.0～5.0 | 0 | 2475 | 0.942 |
|  | 5.0～13.0 | 9 | 46 | 0.018 |
| *U. citrina* | -29～-5.0 | -17 | 8257 | 0.195 |
|  | -5.0～5.0 | 0 | 25098 | 0.592 |
|  | 5.0～11.0 | 8 | 9045 | 0.213 |
| *P. persalinus* | -9.0～-3.00 | -6.0 | 15 | 0.053 |
|  | -3.00～3.00 | 0 | 254 | 0.904 |
|  | 3.00～9.0 | 6.0 | 12 | 0.043 |

**TABLE S3 ENC values of nine ciliates.**

| **Species** | **Class boundary** | **Amount** | **Frequency** |
| --- | --- | --- | --- |
| *S. lemnae* | 20～35 | 175 | 0.009 |
|  | 35～45 | 8105 | 0.397 |
|  | **45～61** | **12142** | **0.594** |
| *T. thermophila* | 20～35 | 2300 | 0.090 |
|  | **35～45** | **21414** | **0.935** |
|  | 45～61 | 1932 | 0.075 |
| *O. trifallax* | 20～35 | 16 | 0.011 |
|  | 35～45 | 434 | 0.234 |
|  | **45～61** | **1027** | **0.695** |
| *P. tetraurelia* | 20～35 | 18 | 0.025 |
|  | 35～45 | 335 | 0.461 |
|  | **45～61** | **373** | **0.514** |
| *P. caudatum* | 20～35 | 23 | 0.030 |
|  | 35～45 | 286 | 0.369 |
|  | **45～61** | **467** | **0.602** |
| *E. octocarinatus* | 20～35 | 0 | 0 |
|  | 35～45 | 10 | 0.098 |
|  | **45～61** | **92** | **0.902** |
| *S. coeruleus* | 20～35 | 2 | 0.0008 |
|  | 35～45 | 169 | 0.0643 |
|  | **45～61** | **2457** | **0.9350** |
| *U. citrina* | 20～35 | 425 | 0.010 |
|  | 35～45 | 5481 | 0.129 |
|  | **45～61** | **36494** | **0.861** |
| *P. persalinus* | 20～35 | 1 | 0.004 |
|  | **35～45** | **261** | **0.929** |
|  | 45～61 | 19 | 0.068 |

**TABLE S4 RSCU values of nine ciliates.**

| **Animo acid** | **Codon** | ***U. citrina*** | ***E. octocarinatus*** | ***S. lemnae*** | ***O. trifallax*** | ***S. coeruleus*** | ***T. thermophila*** | ***P. tetraurelia*** | ***P. caudatum*** | ***P. persalinus*** |
| --- | --- | --- | --- | --- | --- | --- | --- | --- | --- | --- |
| Ter | UGA | 1.00 | 1.00 | 1.00 | 1.00 | 1.00 | 1.00 | 1.00 | 1.00 | 1.00 |
| Trp | UGG | 1.00 | 1.00 | 1.00 | 1.00 | 1.00 | 1.00 | 1.00 | 1.00 | 1.00 |
| Met | AUG | 1.00 | 1.00 | 1.00 | 1.00 | 1.00 | 1.00 | 1.00 | 1.00 | 1.00 |
| Phe | **UUU^***^** | **1.83** | **1.10** | **1.17** | **1.35** | **1.46** | **1.50** | **1.30** | **1.38** | **1.68** |
|  | UUC | 0.73 | 0.90 | 0.83 | 0.65 | 0.54 | 0.50 | 0.70 | 0.62 | 0.32 |
| Tyr | **UAU^***^** | **1.11** | **1.16** | **1.31** | **1.42** | **1.53** | **1.61** | **1.33** | **1.48** | **1.69** |
|  | UAC | 0.64 | 0.84 | 0.69 | 0.58 | 0.47 | 0.39 | 0.67 | 0.52 | 0.31 |
| Lys | **AAA^*^** | 1.10 | 0.86 | 1.17 | 1.24 | 1.43 | **1.24** | **1.71** | **1.40** | **1.57** |
|  | AAG | 0.90 | 1.14 | 0.83 | 0.76 | 0.57 | 0.76 | 0.29 | 0.60 | 0.43 |
| Asp | **GAU^*^** | 0.89 | 1.24 | 1.51 | 1.47 | 1.52 | **1.56** | **1.64** | **1.59** | **1.54** |
|  | GAC | 0.41 | 0.76 | 0.49 | 0.53 | 0.48 | 0.44 | 0.36 | 0.41 | 0.46 |
| His | **CAU^*^** | 0.77 | 1.25 | 1.44 | 1.60 | 1.44 | **2.00** | **1.60** | **1.48** | **1.63** |
|  | CAC | 1.23 | 0.75 | 0.56 | 0.40 | 0.56 | 0.67 | 0.40 | 0.52 | 0.38 |
| Cys | UGU | 1.60 | 0.88 | 0.94 | 1.20 | 1.23 | 1.14 | 1.67 | 1.25 | 1.00 |
|  | UGC | 0.17 | 1.13 | 1.06 | 0.80 | 0.77 | 0.86 | 0.33 | 0.75 | 0.45 |
| Asn | **AAU^*^** | 0.71 | 1.24 | 1.42 | 1.45 | 1.22 | **1.43** | **1.77** | **1.50** | **1.75** |
|  | AAC | 1.29 | 0.76 | 0.58 | 0.55 | 0.63 | 0.57 | 0.23 | 0.50 | 0.25 |
| Glu | **GAA^*^** | 0.98 | 1.20 | 1.24 | 1.26 | 1.42 | **1.60** | **1.89** | **1.40** | **1.44** |
|  | GAG | 0.77 | 0.80 | 0.76 | 0.74 | 0.58 | 0.40 | 0.11 | 0.60 | 0.56 |
| Ile | **AUU^**^** | **1.64** | **1.20** | **1.4** | **1.15** | **1.22** | 1.35 | 0.93 | 1.38 | 1.59 |
|  | AUC | 0.75 | 0.71 | 0.63 | 0.71 | 0.59 | 0.31 | 0.53 | 0.54 | 0.23 |
|  | **AUA^*^** | 1.25 | 1.09 | 0.96 | 1.15 | 1.19 | **1.35** | **1.53** | **1.08** | **1.19** |
| Gln | **UAA^**^** | **1.14** | **1.45** | **1.55** | **1.76** | **1.70** | 0.89 | 0.78 | 2.01 | 2.52 |
|  | UAG | 0.07 | 0.58 | 0.87 | 0.48 | 0.72 | 0.65 | 0.72 | 0.70 | 0.77 |
|  | **CAA^**^** | **1.41** | **1.33** | **1.08** | **1.28** | **1.10** | 1.00 | 1.63 | 0.93 | 0.47 |
|  | CAG | 1.51 | 0.64 | 0.50 | 0.48 | 0.48 | 1.00 | 0.22 | 0.36 | 0.23 |
| Gly | GGU | 0.47 | 1.14 | 1.15 | 0.67 | 1.13 | 0.47 | 1.00 | 1.16 | 1.20 |
|  | GGC | 0.71 | 0.86 | 0.69 | 1.17 | 0.51 | 0.47 | 0.26 | 0.57 | 0.46 |
|  | **GGA^*^** | 1.41 | 0.86 | 1.76 | 1.83 | 1.53 | **3.06** | **3.00** | **1.71** | **1.50** |
|  | GGG | 0.10 | 1.14 | 0.4 | 0.33 | 0.83 | 0.62 | 0.77 | 0.56 | 0.83 |
| Val | **GUU^***^** | **1.52** | **1.17** | **1.53** | **1.14** | **1.63** | **2.13** | **1.67** | **1.56** | **1.90** |
|  | GUC | 0.95 | 0.50 | 0.67 | 0.57 | 0.53 | 0.50 | 0.67 | 0.48 | 0.54 |
|  | GUA | 0.95 | 1.50 | 1.19 | 1.14 | 1.18 | 0.88 | 1.00 | 1.24 | 1.11 |
|  | GUG | 0.57 | 0.83 | 0.62 | 1.14 | 0.66 | 0.50 | 0.67 | 0.71 | 0.45 |
| Pro | **CCU^***^** | **1.68** | **1.45** | **1.58** | **1.50** | **1.46** | **1.67** | **1.60** | **1.51** | **1.59** |
|  | CCC | 0.65 | 0.43 | 0.43 | 0.50 | 0.80 | 0.33 | 2.00 | 0.53 | 0.50 |
|  | **CCA^***^** | **1.55** | **1.09** | **1.82** | **2.00** | **1.52** | **2.00** | **2.00** | **1.81** | **1.42** |
|  | CCG | 0.13 | 0.36 | 0.17 | 0.02 | 0.23 | 0.57 | 0.50 | 0.15 | 0.49 |
| Thr | ACU | 0.64 | 1.68 | 1.95 | 1.33 | 1.53 | 2.00 | 1.54 | 1.49 | 0.50 |
|  | ACC | 0.29 | 0.52 | 0.54 | 0.89 | 0.75 | 0.38 | 0.62 | 0.67 | 0.50 |
|  | **ACA^*^** | 0.07 | 1.68 | 1.35 | 1.33 | 1.47 | **1.63** | **1.54** | **1.66** | **1.35** |
|  | ACG | 0.65 | 0.13 | 0.16 | 0.44 | 0.25 | 0.10 | 0.31 | 0.19 | 0.34 |
| Ala | **GCU^*^** | 0.57 | 2.10 | 1.86 | 1.41 | 1.73 | **2.25** | **1.71** | **1.44** | **1.56** |
|  | GCC | 1.71 | 0.57 | 0.55 | 0.86 | 0.57 | 0.43 | 0.57 | 0.59 | 0.63 |
|  | **GCA^***^** | **1.14** | **1.33** | **1.43** | **1.62** | **1.39** | **1.50** | **1.14** | **1.80** | **1.27** |
|  | GCG | 0.80 | 0.21 | 0.16 | 0.11 | 0.32 | 0.25 | 0.57 | 0.17 | 0.54 |
| Arg | **AGA^***^** | **1.31** | **3.63** | **4.40** | **4.74** | **2.90** | **3.75** | **3.00** | **3.46** | **2.87** |
|  | **AGG^*^** | 2.44 | 1.74 | 0.95 | 0.32 | 1.42 | **1.50** | **2.00** | **1.48** | **1.48** |
|  | CGU | 0.38 | 0.16 | 0.19 | 0.49 | 0.47 | 0.75 | 1.00 | 0.30 | 0.47 |
|  | CGC | 0.56 | 0.12 | 0.12 | 0.16 | 0.29 | 0.44 | 0.14 | 0.12 | 0.22 |
|  | CGA | 0.75 | 0.35 | 0.29 | 0.07 | 0.69 | 0.27 | 0.58 | 0.52 | 0.66 |
|  | CGG | 0.56 | 0.18 | 0.05 | 0.01 | 0.23 | 0.29 | 0.13 | 0.12 | 0.29 |
| Leu | **UUA^*^** | 0.58 | 1.27 | 1.55 | 1.50 | 2.01 | **2.34** | **2.18** | **2.24** | **3.10** |
|  | UUG | 1.55 | 0.64 | 1.06 | 1.63 | 1.08 | 1.32 | 0.91 | 1.16 | 1.10 |
|  | **CUU^**^** | **1.74** | **1.36** | **1.39** | **1.25** | **1.27** | 0.92 | 1.82 | 1.03 | 0.77 |
|  | CUC | 0.58 | 0.73 | 0.57 | 0.38 | 0.43 | 0.41 | 0.91 | 0.42 | 0.15 |
|  | CUA | 0.97 | 0.91 | 0.99 | 0.75 | 0.77 | 0.81 | 0.91 | 0.72 | 0.60 |
|  | CUG | 0.58 | 1.09 | 0.44 | 0.50 | 0.43 | 0.20 | 0.18 | 0.42 | 0.30 |
| Ser | **UCU^*^** | 1.04 | 1.63 | 1.48 | 0.97 | 1.48 | **1.71** | **1.45** | **1.51** | **1.25** |
|  | UCC | 0.91 | 0.41 | 0.42 | 0.39 | 0.71 | 0.21 | 0.41 | 0.76 | 0.61 |
|  | UCA | 1.43 | 0.92 | 1.76 | 2.32 | 1.58 | 0.86 | 2.07 | 1.51 | 0.84 |
|  | UCG | 0.91 | 0.61 | 0.22 | 0.39 | 0.31 | 0.21 | 0.15 | 0.25 | 0.42 |
|  | **AGU^***^** | **1.76** | **1.33** | **1.32** | **1.55** | **1.10** | **1.61** | **1.66** | **1.28** | **2.19** |
|  | AGC | 1.24 | 1.20 | 0.80 | 0.55 | 0.81 | 1.39 | 0.41 | 0.69 | 0.69 |

Note: RSCU, the relative synonymous codon usage；

*** represents the codon commonly preferred by nine ciliates;

** represents the codon commonly preferred by the four ciliates of Spirotrichea;

* represents the codon commonly preferred by the four ciliates of Oligohymenophorea;

**TABLE S5-1 Correlation coefficients of CUB indices in *E. octocarinatus*.**

|  | T3s | C3s | A3s | G3s | ENC | GC3s | GC12 | L-sym | L-aa |
| --- | --- | --- | --- | --- | --- | --- | --- | --- | --- |
| T3s |  |  |  |  |  |  |  |  |  |
| C3s | -0.261** |  |  |  |  |  |  |  |  |
| A3s | -0.330^**^ | -0.177 |  |  |  |  |  |  |  |
| G3s | -0.414^**^ | -0.199^*^ | -0.329^**^ |  |  |  |  |  |  |
| ENC | -0.147 | 0.002 | -0.385^**^ | 0.506^**^ |  |  |  |  |  |
| GC3s | -0.603^**^ | 0.588^**^ | -0.464^**^ | 0.636^**^ | 0.429^**^ |  |  |  |  |
| GC12 | -0.269^**^ | 0.326^**^ | -0.258^**^ | 0.110 | 0.224^*^ | 0.445^**^ |  |  |  |
| L-sym | -0.004 | -0.054 | -0.220^*^ | 0.158 | 0.209^*^ | 0.119 | 0.027 |  |  |
| L-aa | -0.013 | -0.055 | -0.225^*^ | 0.171 | 0.211^*^ | 0.130 | 0.027 | 1.000^**^ |  |

Note: **means extremely significant correlation (*P*＜0.01)；*means significant correlation (*P*＜0.05).

**TABLE S5-2 Correlation coefficients of CUB indices in *S. lemnae.***

|  | T3s | C3s | A3s | G3s | ENC | GC3s | GC12 | L-sym | L-aa |
| --- | --- | --- | --- | --- | --- | --- | --- | --- | --- |
| T3s |  |  |  |  |  |  |  |  |  |
| C3s | -0.720^**^ |  |  |  |  |  |  |  |  |
| A3s | 0.292^**^ | -0.620^**^ |  |  |  |  |  |  |  |
| G3s | -0.238^**^ | 0.127^**^ | -0.372^**^ |  |  |  |  |  |  |
| ENC | -0.008 | -0.001 | 0.003 | -0.007 |  |  |  |  |  |
| GC3s | -0.008 | 0.019^**^ | -0.020^**^ | -0.001 | 0.009 |  |  |  |  |
| GC12 | -0.009 | 0.011 | -0.01 | -0.001 | 0.018^*^ | 0.711^**^ |  |  |  |
| L-sym | -0.007 | 0.004 | 0.007 | -0.004 | -0.008 | -0.056^**^ | -0.093^**^ |  |  |
| L-aa | -0.007 | 0.005 | 0.007 | -0.004 | -0.008 | -0.054^**^ | -0.091^**^ | 1.000^**^ |  |

Note: **means extremely significant correlation (*P*＜0.01)；*means significant correlation (*P*＜0.05).

**TABLE S5-3 Correlation coefficients of CUB indices in *T. thermophila.***

|  | T3s | C3s | A3s | G3s | ENC | GC3s | GC12 | L-sym | L-aa |
| --- | --- | --- | --- | --- | --- | --- | --- | --- | --- |
| T3s |  |  |  |  |  |  |  |  |  |
| C3s | -0.402^**^ |  |  |  |  |  |  |  |  |
| A3s | -0.121^**^ | -0.564^**^ |  |  |  |  |  |  |  |
| G3s | -0.322^**^ | -0.019^**^ | 0.016^**^ |  |  |  |  |  |  |
| ENC | -0.268^**^ | 0.074^**^ | 0.047^**^ | 0.358^**^ |  |  |  |  |  |
| GC3s | -0.534^**^ | 0.880^**^ | -0.593^**^ | 0.420^**^ | 0.217^**^ |  |  |  |  |
| GC12 | -0.151^**^ | 0.583^**^ | -0.676^**^ | -0.186^**^ | -0.065^**^ | 0.533^**^ |  |  |  |
| L-sym | 0.096^**^ | -0.087^**^ | 0.059^**^ | -0.049^**^ | 0.027^**^ | -0.069^**^ | -0.040^**^ |  |  |
| L-aa | 0.086^**^ | -0.067^**^ | 0.053^**^ | -0.060^**^ | 0.037^**^ | -0.089^**^ | -0.050^**^ | 1.000^**^ |  |

Note: **means extremely significant correlation (*P*＜0.01)；*means significant correlation (*P*＜0.05).

**TABLE S5-4 Correlation coefficients of CUB indices in *O. trifallax.***

|  | T3s | C3s | A3s | G3s | ENC | GC3s | GC12 | L-sym | L-aa |
| --- | --- | --- | --- | --- | --- | --- | --- | --- | --- |
| T3s |  |  |  |  |  |  |  |  |  |
| C3s | -0.760^**^ |  |  |  |  |  |  |  |  |
| A3s | 0.419^**^ | -0.685^**^ |  |  |  |  |  |  |  |
| G3s | -0.238^**^ | 0.147^**^ | -0.309^**^ |  |  |  |  |  |  |
| ENC | -0.083^**^ | -0.03 | -0.057^*^ | 0.380^**^ |  |  |  |  |  |
| GC3s | -0.786^**^ | -0.905^**^ | -0.782^**^ | 0.515^**^ | 0.131^**^ |  |  |  |  |
| GC12 | -0.666^**^ | 0.728^**^ | -0.746^**^ | 0.113^**^ | -0.004 | 0.750^**^ |  |  |  |
| L-sym | 0.161^**^ | -0.133^**^ | 0.080^**^ | -0.081^**^ | -0.035 | -0.153^**^ | -0.155^**^ |  |  |
| L-aa | 0.160^**^ | -0.132^**^ | 0.078^**^ | -0.081^**^ | -0.035 | -0.152^**^ | -0.154^**^ | 1.000^**^ |  |

Note: **means extremely significant correlation (*P*＜0.01)；*means significant correlation (*P*＜0.05).

**TABLE S5-5 Correlation coefficients of CUB indices in *P. tetraurelia.***

|  | T3s | C3s | A3s | G3s | ENC | GC3s | GC12 | L-sym | L-aa |
| --- | --- | --- | --- | --- | --- | --- | --- | --- | --- |
| T3s |  |  |  |  |  |  |  |  |  |
| C3s | -0.607^**^ |  |  |  |  |  |  |  |  |
| A3s | -0.152^**^ | -0.293^**^ |  |  |  |  |  |  |  |
| G3s | -0.195^**^ | 0.007 | -0.461^**^ |  |  |  |  |  |  |
| ENC | -0.202^**^ | 0.383^**^ | -0.488^**^ | 0.471^**^ |  |  |  |  |  |
| GC3s | -0.656^**^ | 0.784^**^ | -0.539^**^ | 0.599^**^ | 0.588^**^ |  |  |  |  |
| GC12 | -0.436^**^ | 0.353^**^ | -0.343^**^ | 0.197^**^ | 0.376^**^ | 0.506^**^ |  |  |  |
| L-sym | -0.032 | 0.056 | -0.09 | 0.027 | 0.049 | 0.062 | 0.101^**^ |  |  |
| L-aa | -0.052 | 0.066 | -0.07 | 0.022 | 0.045 | 0.072 | 0.101^**^ | 1.000^**^ |  |

Note: **means extremely significant correlation (*P*＜0.01)；*means significant correlation (*P*＜0.05).

**TABLE S5-6 Correlation coefficients of CUB indices in *P. caudatum.***

|  | T3s | C3s | A3s | G3s | ENC | GC3s | GC12 | L-sym | L-aa |
| --- | --- | --- | --- | --- | --- | --- | --- | --- | --- |
| T3s |  |  |  |  |  |  |  |  |  |
| C3s | -0.601^**^ |  |  |  |  |  |  |  |  |
| A3s | -0.140^**^ | -0.265^**^ |  |  |  |  |  |  |  |
| G3s | -0.182^**^ | -0.014 | -0.490^**^ |  |  |  |  |  |  |
| ENC | -0.193^**^ | 0.343^**^ | -0.503^**^ | 0.489^**^ |  |  |  |  |  |
| GC3s | -0.619^**^ | 0.738^**^ | -0.579^**^ | 0.636^**^ | 0.591^**^ |  |  |  |  |
| GC12 | -0.402^**^ | 0.361^**^ | -0.454^**^ | 0.222^**^ | 0.391^**^ | 0.538^**^ |  |  |  |
| L-sym | -0.03 | 0.09 | -0.067 | 0.027 | 0.071^*^ | 0.075 | 0.089^*^ |  |  |
| L-aa | -0.08 | 0.06 | -0.058 | 0.035 | 0.062^*^ | 0.069 | 0.079^*^ | 1.000^**^ |  |

Note: **means extremely significant correlation (*P*＜0.01)；*means significant correlation (*P*＜0.05).

**TABLE S5-7 Correlation coefficients of CUB indices in *S. coeruleus.***

|  | T3s | C3s | A3s | G3s | ENC | GC3s | GC12 | L-sym | L-aa |
| --- | --- | --- | --- | --- | --- | --- | --- | --- | --- |
| T3s |  |  |  |  |  |  |  |  |  |
| C3s | -0.006 |  |  |  |  |  |  |  |  |
| A3s | 0.103^**^ | -0.01 |  |  |  |  |  |  |  |
| G3s | -0.259^**^ | -0.245^**^ | -0.357^**^ |  |  |  |  |  |  |
| ENC | -0.300^**^ | -0.083^**^ | -0.392^**^ | 0.480^**^ |  |  |  |  |  |
| GC3s | -0.025 | 0.249^**^ | -0.041^*^ | -0.466^**^ | 0.094^**^ |  |  |  |  |
| GC12 | -0.029 | 0.223^**^ | -0.045^*^ | -0.069^**^ | 0.180^**^ | 0.781^**^ |  |  |  |
| L-sym | -0.340^**^ | -0.026 | -0.468^**^ | -0.049^**^ | 0.142^**^ | -0.085^**^ | -0.052^**^ |  |  |
| L-aa | -0.220^**^ | -0.017 | -0.248^**^ | -0.052^**^ | 0.143^**^ | -0.065^**^ | -0.073^**^ | 1.000^**^ |  |

Note: **means extremely significant correlation (*P*＜0.01)；*means significant correlation (*P*＜0.05).

**TABLE S5-8 Correlation coefficients of CUB indices in *U. citrina.***

|  | T3s | C3s | A3s | G3s | ENC | GC3s | GC12 | L-sym | L-aa |
| --- | --- | --- | --- | --- | --- | --- | --- | --- | --- |
| T3s |  |  |  |  |  |  |  |  |  |
| C3s | -0.458^**^ |  |  |  |  |  |  |  |  |
| A3s | -0.219^**^ | -0.321^**^ |  |  |  |  |  |  |  |
| G3s | -0.345^**^ | -0.111^**^ | -0.305^**^ |  |  |  |  |  |  |
| ENC | -0.218^**^ | 0.252^**^ | -0.166^**^ | 0.189^**^ |  |  |  |  |  |
| GC3s | -0.640^**^ | 0.689^**^ | -0.546^**^ | 0.604^**^ | 0.328^**^ |  |  |  |  |
| GC12 | -0.399^**^ | 0.284^**^ | -0.377^**^ | 0.238^**^ | 0.270^**^ | 0.513^**^ |  |  |  |
| L-sym | -0.056^**^ | 0.051^**^ | -0.059^**^ | 0.038^**^ | 0.275^**^ | 0.079^**^ | 0.147^**^ |  |  |
| L-aa | -0.062^**^ | 0.049^**^ | -0.060^**^ | 0.047^**^ | 0.277^**^ | 0.085^**^ | 0.150^**^ | 1.000^**^ |  |

Note: **means extremely significant correlation (*P*＜0.01)；*means significant correlation (*P*＜0.05).

**TABLE S5-9 Correlation coefficients of CUB indices in *P. persalinus.***

|  | T3s | C3s | A3s | G3s | ENC | GC3s | GC12 | L-sym | L-aa |
| --- | --- | --- | --- | --- | --- | --- | --- | --- | --- |
| T3s |  |  |  |  |  |  |  |  |  |
| C3s | -0.774^**^ |  |  |  |  |  |  |  |  |
| A3s | -0.273^**^ | -0.025 |  |  |  |  |  |  |  |
| G3s | 0.140^*^ | -0.344^**^ | -0.600^**^ |  |  |  |  |  |  |
| ENC | -0.367^**^ | 0.563^**^ | -0.398^**^ | 0.088 |  |  |  |  |  |
| GC3s | -0.646^**^ | 0.717^**^ | -0.507^**^ | 0.397^**^ | 0.610^**^ |  |  |  |  |
| GC12 | -0.398^**^ | 0.444^**^ | -0.564^**^ | 0.366^**^ | 0.521^**^ | 0.762^**^ |  |  |  |
| L-sym | 0.045 | -0.028 | -0.005 | 0.002 | 0.004 | -0.033 | -0.066 |  |  |
| L-aa | 0.040 | -0.029 | -0.005 | 0.004 | 0.003 | -0.033 | -0.066 | 1.000^**^ |  |

Note: **means extremely significant correlation (*P*＜0.01)；*means significant correlation (*P*＜0.05).

**TABLE S6 List of species for phylogeny analysis based on SSU rDNA.**

| **Number** | **Class** | **Subclass** | **Order** | **Family** | **Taxon** | **GenBank accession numbers** |
| --- | --- | --- | --- | --- | --- | --- |
| 1 | ARMOPHOREA |  | Clevelandellida | Clevelandellidae | *Clevelandella panesthiae* | KC139719 |
| 2 | ARMOPHOREA |  | Clevelandellida | Nyctotheridae | *Nyctotherus ovalis* | AJ222678 |
| 3 | COLPODEA |  | Bryometopida | Bryometopida | *Bryometopus atypicus* | EU039886 |
| 4 | COLPODEA |  | Bryophryida | Bryophryidae | *Notoxoma parabryophryides* | EU039903 |
| 5 | COLPODEA |  | Bursariomorphida | Bursariidae | *Bursaria truncatella* | U82204 |
| 6 | COLPODEA |  | Colpodida | Hausmanniellidae | *Bresslauides discoideus* | HM140394 |
| 7 | HETEROTRICHEA |  | Heterotrichida | Climacostomidae | *Climacostomum virens* | EU583990 |
| 8 | HETEROTRICHEA |  | Heterotrichida | Climacostomidae | *Fabrea salina* | KM222110 |
| 9 | HETEROTRICHEA |  | Heterotrichida | Condylostomatidae | *Condylostoma magnum* | KM222108 |
| 10 | HETEROTRICHEA |  | Heterotrichida | Folliculinidae | *Folliculina simplex* | EU583992 |
| 11 | HETEROTRICHEA |  | Heterotrichida | Peritromidae | *Peritromus faurei* | EU583993 |
| 12 | HETEROTRICHEA |  | Heterotrichida | Spirostomidae | *Anigsteinia clarissima* | KM222109 |
| 13 | HETEROTRICHEA |  | Heterotrichida | Spirostomidae | *Spirostomum sp.* | FJ998027 |
| 14 | HETEROTRICHEA |  | Heterotrichida | Stentoridae | *Stentor sp.* | KM222111 |
| 15 | [HETEROTRIC](https://www.ncbi.nlm.nih.gov/taxonomy/194287)HEA |  | [Heterotrichida](https://www.ncbi.nlm.nih.gov/taxonomy/5957) | [Stentoridae](https://www.ncbi.nlm.nih.gov/taxonomy/219169) | *Stentor coeruleus* | LT628482 |
| 16 | KARYORELICTEA |  | Protostomatida | Trachelocercidae | *Trachelocerca sagitta* | KC542935 |
| 17 | LITOSTOMATEA | Haptoria | Pleurostomatida | Amphileptidae | *Amphileptus songi* | FJ876974 |
| 18 | LITOSTOMATEA | Haptoria | Pleurostomatida | Amphileptidae | *Epiphyllum shenzhenense* | GU574809 |
| 19 | LITOSTOMATEA | Trichostomatia | Entodiniomorphida | Troglodytellidae | *Troglodytella abrassarti* | AB437346 |
| 20 | LITOSTOMATEA | Trichostomatia | Vestibuliferida | Balantidiidae | *Balantidium entozoon* | EU581716 |
| 21 | NASSOPHOREA |  | Microthoracida | Leptopharyngidae | *Leptopharynx costatus* | EU286811 |
| 22 | NASSOPHOREA |  | Nassulida | Nassulidae | *Nassula labiata* | KC832949 |
| 23 | OLIGOHYMENOPHOREA | Apostomatia | Apostomatida | Colliniidae | *Pseudocollinia beringensis* | HQ591474 |
| 24 | OLIGOHYMENOPHOREA | Astomatia | Astomatida | Anoplophryidae | *Almophrya bivacuolata* | HQ446281 |
| 25 | OLIGOHYMENOPHOREA | Hymenostomatia | Ophryoglenida | Ichthyophthiriidae | *Ichthyophthirius multifiliis* | U17354 |
| 26 | OLIGOHYMENOPHOREA | Hymenostomatia | Tetrahymenida | Tetrahymenidae | *Tetrahymena thermophila* | X56165 |
| 27 | OLIGOHYMENOPHOREA | Peniculia | Peniculida | Parameciidae | *Paramecium tetraurelia* | AY102613 |
| 28 | OLIGOHYMENOPHOREA | Peniculia | Urocentrida | Urocentridae | *Urocentrum turbo* | AF255357 |
| 29 | OLIGOHYMENOPHOREA | Peritrichia | Mobilida | Trichodinidae | *Trichodina heterodentata* | AY788099 |
| 30 | OLIGOHYMENOPHOREA | Peritrichia | Mobilida | Trichodinidae | *Trichodinella myakkae* | AY102176 |
| 31 | OLIGOHYMENOPHOREA | Scuticociliatia | Loxocephalida | Cinetochilidae | *Cinetochilum ovale* | FJ870103 |
| 32 | OLIGOHYMENOPHOREA | Scuticociliatia | Loxocephalida | Cinetochilidae | *Pseudoplatynematum denticulatum* | JX310020 |
| 33 | OLIGOHYMENOPHOREA | Scuticociliatia | Loxocephalida | Cinetochilidae | *Sathrophilus holtae* | FJ868188 |
| 34 | OLIGOHYMENOPHOREA | Scuticociliatia | Loxocephalida | Loxocephalidae | *Cardiostomatella vermiformis* | AY881632 |
| 35 | OLIGOHYMENOPHOREA | Scuticociliatia | Loxocephalida | Loxocephalidae | *Paratetrahymena wassi* | GQ292767 |
| 36 | OLIGOHYMENOPHOREA | Scuticociliatia | Philasterida | Orchitophryidae | *Metanophrys sinensis* | HM236336 |
| 37 | OLIGOHYMENOPHOREA | Scuticociliatia | Philasterida | Parauronematidae | *Miamiensis avidus* | JN885091 |
| 38 | OLIGOHYMENOPHOREA | Scuticociliatia | Philasterida | Philasteridae | *Philasterides armatalis* | FJ848877 |
| 39 | OLIGOHYMENOPHOREA | Scuticociliatia | Philasterida | Pseudocohnilembidae | *Pseudocohnilembus hargisi* | JN885090 |
| 40 | OLIGOHYMENOPHOREA | Scuticociliatia | Philasterida | Uronematidae | *Uronema marinum* | GQ465466 |
| 41 | OLIGOHYMENOPHOREA | Scuticociliatia | Philasterida | Orchitophryidae | *Mesanophrys carcini* | JN885086 |
| 42 | OLIGOHYMENOPHOREA | [Scuticociliatia](https://www.ncbi.nlm.nih.gov/taxonomy/35094) | [Philasterida](https://www.ncbi.nlm.nih.gov/taxonomy/198613) | [Pseudocohnilembidae](https://www.ncbi.nlm.nih.gov/taxonomy/35102) | *Pseudocohnilembus persalinus* | GU584096 |
| 43 | PROSTOMATEA |  | Prorodontida | Placidae | *Placus salinus* | KC832954 |
| 44 | PROSTOMATEA |  | Prorodontida | Prorodontidae | *Prorodon ovum* | KM222104 |
| 45 | PROTOCRUZIEA |  | Protocruziida | Protocruziidae | *Protocruzia contrax* | DQ190467 |
| 46 | SPIROTRICHEA | Choreotrichia | Tintinnida | Codonellidae | *Tintinnopsis tubulosoides* | AF399111 |
| 47 | SPIROTRICHEA | Choreotrichia | Tintinnida | Metacylididae | *Helicostomella subulata* | JQ716991 |
| 48 | SPIROTRICHEA | Hypotrichia | Discocephalida | Discocephalidae | *Paradiscocephalus elongatus* | EU684746 |
| 49 | SPIROTRICHEA | Hypotrichia | Discocephalida | Discocephalidae | *Pseudoamphisiella quadrinucleata* | EU518416 |
| 50 | SPIROTRICHEA | Hypotrichia | Euplotida | Aspidiscidae | *Aspidisca leptaspis* | EU880597 |
| 51 | SPIROTRICHEA | Hypotrichia | Euplotida | Certesiidae | *Certesia quadrinucleta* | KM222097 |
| 52 | SPIROTRICHEA | Hypotrichia | Euplotida | Euplotidae | *Euplotes encysticus* | FJ346569 |
| 53 | SPIROTRICHEA | Hypotrichia | Euplotida | Euplotidae | *Euplotes sinicus* | FJ876980 |
| 54 | SPIROTRICHEA | Hypotrichia | Euplotida | Gastrocirrhidae | *Gastrocirrhus monilifer* | DQ864734 |
| 55 | SPIROTRICHEA | Hypotrichia | Euplotida | Uronychiidae | *Diophrys parappendiculata* | EU267928 |
| 56 | SPIROTRICHEA | Hypotrichia | Euplotida | Uronychiidae | *Uronychia sinica* | FJ876982 |
| 57 | SPIROTRICHEA | Stichotrichia | Sporadotrichida | Oxytrichidae | *Hemigastrostyla enigmatica* | FJ870096 |
| 58 | SPIROTRICHEA | Stichotrichia | Sporadotrichida | Oxytrichidae | *Oxytricha granulifera* | AF508762 |
| 59 | SPIROTRICHEA | Stichotrichia | Sporadotrichida | Oxytrichidae | *Tachysoma pellionellum* | KM222096 |
| 60 | SPIROTRICHEA | Stichotrichia | Sporadotrichida | Oxytrichidae | *Tetmemena puslata* | KM222092 |
| 61 | SPIROTRICHEA | Stichotrichia | Sporadotrichida | Oxytrichidae | *Oxytricha trifallax* | AF198110 |
| 62 | SPIROTRICHEA | Stichotrichia | Urostylida | Pseudokeronopsidae | *Nothoholosticha fasciola* | FJ377548 |
| 63 | SPIROTRICHEA | Stichotrichia | Urostylida | Pseudokeronopsidae | *Pseudokeronopsis flava* | DQ227798 |
| 64 | SPIROTRICHEA | Stichotrichia | Urostylida | Pseudokeronopsidae | *Thigmokeronopsis stoecki* | EU220226 |
| 65 | SPIROTRICHEA | Stichotrichia | Urostylida | Pseudokeronopsidae | *Uroleptopsis citrina* | FJ870094 |
| 66 | SPIROTRICHEA | Stichotrichia | Urostylida | Pseudourostylidae | *Pseudourostyla sp.* | FJ775725 |
| 67 | SPIROTRICHEA | Stichotrichia | [Sporadotrichida](https://www.ncbi.nlm.nih.gov/taxonomy/693921) | [Oxytrichidae](https://www.ncbi.nlm.nih.gov/taxonomy/57506) | *Stylonychia lemnae* | HQ432920 |
| 68 | SPIROTRICHEA | Hypotrichia | Euplotida | Euplotidae | *Euplotes octocarinatus* | LT623905 |

**S1 Perl scripts**

**Perl 1:** **Remove the newline characters in the sequence**;

#!/usr/bin/env perl

#===============================================================================

#

# FILE: 11.pl

#

# USAGE:./11.pl

#

#DESCRIPTION: Remove the newline characters in the sequence

#

# OPTIONS: ---

# REQUIREMENTS: ---

# BUGS: ---

# NOTES: ---

# AUTHOR: YAN BAI (YanB), baiyan789@163.com

# ORGANIZATION:

# VERSION: 1.0

# CREATED: 2019/6/20 13:47:51

# REVISION: ---

#===============================================================================

while(<>){

chomp;

if(/>/){

print "\n$_\n";

}else{

print $_;}}

**Perl 2: Retain sequences with a sequence length> 300bp; remove repeated sequences; remove reverse complementary sequences;**

#!/usr/bin/env perl

#===============================================================================

#

# FILE: 1.pl

#

# USAGE: ./1.pl

#

# DESCRIPTION: Retain sequences with a sequence length> 300bp; remove repeated sequences; remove reverse complementary sequences

#

#

# OPTIONS: ---

# REQUIREMENTS: ---

# BUGS: ---

# NOTES: ---

# AUTHOR: YOUR NAME (),

# ORGANIZATION:

# VERSION: 1.0

# CREATED: 2019/6/17 23:05:33

# REVISION: ---

#===============================================================================

while(<>){

chomp;

if(/>/){

$a = $_;}

else{

$m = reverse $_;

$m=~tr/ATGC/TACG/;

$re{$m} = 1;

if($re{$_}){}else{

$ha{$_} = $a;}}}

foreach (keys %ha){

$n = length($_);

if($n>=300){

print "$ha{$_}\n$_\n";}

}

#print %ha;

**Perl 3: The sequence is incomplete and there is a stop codon in the middle of the sequence is deleted, and the CDS containing the start codon ATG and the end stop codons UAA, UAG, UGA is retained**;

#!/usr/bin/perl

while ( <> ) {

chomp;

if(/>/){

$a=$_;

}else{$m = $_;

@p = $m =~ /(.{3})/g;

@b = grep s/ATG|TGG|TAA|TAG|TGA//,@p;

$z = join ('',@p);

$ha{$a} =$z;}}

foreach (keys %ha){

print"$_\n$ha{$_}\n";}
